# A Double-Branch Surface Detection System for Armatures in Vibration Motors with Miniature Volume Based on ResNet-101 and FPN

**DOI:** 10.3390/s20082360

**Published:** 2020-04-21

**Authors:** Tao Feng, Jiange Liu, Xia Fang, Jie Wang, Libin Zhou

**Affiliations:** 1 School of Mechanical Engineering Sichuan University, Chengdu 610041, Sichuan, Chinaliujiange666@163.com (J.L.); wangjie@scu.edu.cn (J.W.); 2 College of Letters and Science, University of Wisconsin Madison, Madison, WI 53707, USA; Lzhou228@wisc.edu

**Keywords:** armature, computer vision, deep learning, surface inspection

## Abstract

In this paper, a complete system based on computer vision and deep learning is proposed for surface inspection of the armatures in a vibration motor with miniature volume. A device for imaging and positioning was designed in order to obtain the images of the surface of the armatures. The images obtained by the device were divided into a training set and a test set. With continuous experimental exploration and improvement, the most efficient deep-network model was designed. The results show that the model leads to high accuracy on both the training set and the test set. In addition, we proposed a training method to make the network designed by us perform better. To guarantee the quality of the motor, a double-branch discrimination mechanism was also proposed. In order to verify the reliability of the system, experimental verification was conducted on the production line, and a satisfactory discrimination performance was reached. The results indicate that the proposed detection system for the armatures based on computer vision and deep learning is stable and reliable for armature production lines.

## 1. Introduction

A vibration motor is a source of excitation. Small vibration motors are used in digital products like cell phones to provide a vibration sense. Large vibration motors are used in metallurgy and mining to screen ingredients [1]. In terms of the vibration motors used in digital products, the quality of the motor is an important factor that has an impact on the user experience. In the process of motor production, the armature is assembled into a shell with magnets and bearings [2], so incipient faults in any part of the machinery could produce a chain reaction and lead to its defects [3,4,5]. However, due to its miniature volume, it is difficult to detect these defects.

Much effort has been made in fault diagnosis of rotating machinery. Asr et al. [6] designed a feature extraction method using empirical mode decomposition and fed the extracted features into a non-native Bayesian classifier for intelligent fault diagnosis of rotating machinery. Georgoulas et al. [7] applied symbolic dynamic entropy features to extract features of gearbox signals and applied a support vector machine to recognize the health conditions.

However, the inspection conducted after assembly is always dramatically influenced by the mechanical system. It is difficult to explore the form of the signal. Therefore, each part should be carefully examined before assembly. For a vibration motor, the armature is the core component. If the quality of the armature is ensured, many problems will be avoided during motor rotation. In the traditional way, surface inspection of the armature is done manually, which is costly and can be easily disturbed by many subjective factors [8]. Therefore, it is important for a factory to apply intelligent inspection to detect the surface of the armature.

With the rapid development of computer vision, more and more image technologies have been used in industrial environments to detect surface quality. Cabral et al. [9] realized the intelligent detection of various glass products by traditional computer vision methods such as edge detection operator and Hough circle. In reference [10], image processing algorithms including Canny edge extraction, histogram equalization, and image morphology closed operation are utilized to extract and locate a joint contour in a complicated background image. Firmin et al. [11] proposed a novel method for detection and quantification of corrosion on a pipe using digital image processing techniques to extract saturation value. All of these are the applications of traditional computer vision, which can only achieve some simple industrial applications. It is difficult to use these applications for the detection of complex industrial objects while obtaining good results.

Therefore, with the development of hardware performance, the deep learning algorithm represented by convolutional neural networks (CNNs) has gradually become the core of the machine learning algorithm [12]. To date, image feature extraction by a CNN has shown great success in the process of image classification [13,14], object detection [15], semantic segmentation [16,17], and high-resolution image reconstruction [18]. A CNN has also been successfully applied to inspect industrial surfaces recently. Masci et al. [19] adopted max-pooling convolutional neural networks to detect the defects in steel. Song et al. [20] proposed a novel residual squeeze-and-excitation network to discover anomalies and inspect the quality of adhesives on battery cell surfaces. Weimer et al. [21] discussed the structural design of the CNN to realize automatic feature extraction in industrial surfaces and verified the proposed elements through the dataset of industrial surface defects.

For surface inspection in this paper, we consider the armatures in micro vibration motors with miniature volume. Considering the rough surface and tiny features, we used more abundant semantic features extracted by the CNN network to detect the surface. The inspection standard comes from the armature manufacturer.

The rest of this paper is organized as follows. Firstly, we introduce the locating device we designed to take photos of the armature. Then, we discuss the method, in which the collected images of armatures were divided into a training set and a test set, and we introduce the classification standard. Finally, we propose a network structure designed for our dataset and a training method to make the network performs better. 

## 2. Related Works

### 2.1. Image Acquisition 

**Figure 1:**
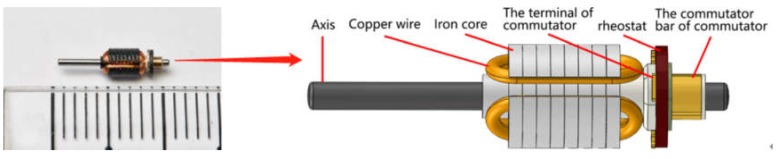
The inspected armature.

Figure 1 shows the inspected armature. We can see that the armature is made of an iron core wrapped with copper wire. The length of the whole armature is about 1 cm. The entire armature is symmetrical about 120° around the central axis. To detect as few images as possible to reduce the detection time, we can convert the detection of the armature to the detection of three major surfaces.

**Figure 2:**
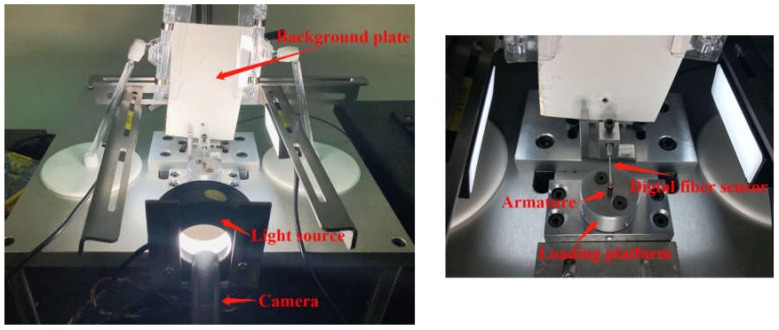
The positioning and imaging device.

In industrial inspection, the workpiece is mostly irregular. In order to reduce the difficulty of positioning and detection, it is necessary to obtain a fixed imaging view through electromechanical structure. Figure 2 depicts the positioning and imaging device. The left side of Figure 2 is the entire device, and the right side is a zoomed-in view of the loading platform. The armature was placed on a loading platform and fixed by a small magnetic shaft hole. The loading platform was driven by a stepper motor to rotate and change the shooting angle of the armature. Because the reflectivity of the copper wire is greater than that of the iron core, when the beam of the digital fiber sensor shines on the iron core, as shown in Figure 1, the amount of reflected light is lower than that in the groove on the iron core shown in Figure 1, which is wrapped with copper wire. We used this phenomenon to find the position of the groove on the iron core. We used this position as the initial position for photographs. Then, taking advantage of the symmetry, a stepper motor with a closed loop system was used to drive the loading platform to precisely rotate 120° two times. In the end, we obtained the photos of three major surfaces, which can reflect the surface defects of a whole armature.

**Figure 3:**
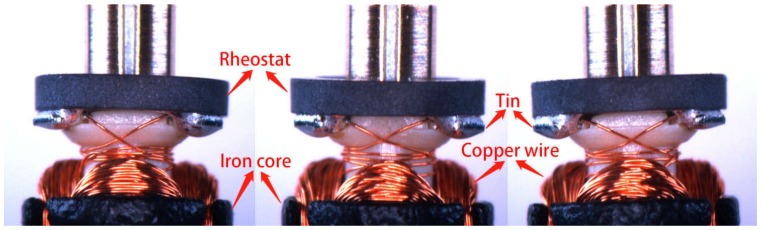
The picture of an armature we obtained from three faces.

Thus, for each armature we obtained three pictures. Whether the armature is defective is determined by detecting the three pictures. To avoid the influence of external illumination, a telephoto lens with fixed focus was selected. The shooting device was an industrial CCD camera with a resolution of 1920 × 960 pixels. The final processed and transmitted image format was JPG with a resolution of 540 × 480. Figure 3 gives the picture of three faces of an armature. From Figure 3, we can see that we obtained a good background and foreground. The edge between the armature and the background is well segmented. We can use simple morphological detection to identify the effective region of the armatures to eliminate unnecessary interference.

### 2.2. Surface Defects on the Armatures

**Figure 4:**
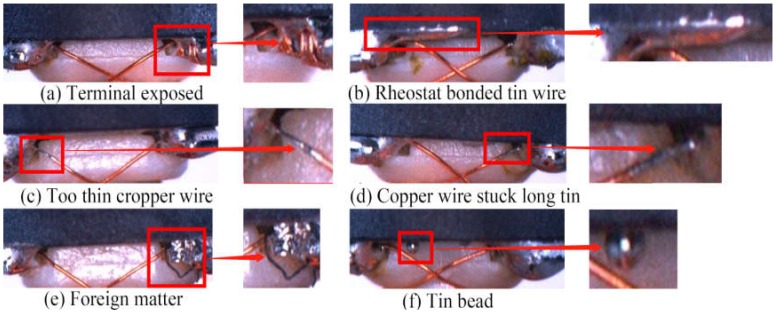
The final inspected area in our study and some of the surface defects.

Figure 3 shows the full perspective of an armature. For our article, we only needed to examine a portion of the image because the pictures taken by the above device had a fixed scale and perspective. Through the template matching algorithm in OpenCV, we could easily match the areas we needed from the template library we created. Figure 4 displays the final inspected area with a resolution of 350 × 120 pixels. We can determine from this figure that there are six kinds of surface defects that need to be inspected. The details of the surface defects are described in Table 1.

### 2.3. Data Calibration and Manual Observation

Each armature with a defective surface had one or more of the above-mentioned defects. Therefore, a defective armature cannot be classified in detail. In the process of labeling, only two classifications were tagged. Positive samples contained no surface defect, negative samples contained surface defects. Photos of the armatures were collected using our shooting and positioning device. We returned the collected photos to the company’s quality control group. The quality control group divided these photos into two categories according to our classification task. Each armature picture was judged separately by two experts. The third expert re-judged the controversial samples. The determination of whether it is a positive sample was based on the judgment of the third expert. This dataset was determined by the quality control team. We regarded it as the correct data classification, which was our ground truth.

Although the inspected area is narrowed, we can see that the negative is still messy and varied. The armatures are tiny, so the defective areas are even smaller. It is a challenge for the eyes to distinguish between good and defective armatures. Due to the small size of the armatures, they must be studied under the microscope, which always contributes to eyestrain and high staff turnover. It is difficult for an enterprise to retain experienced workers. The demand for the enterprise is that the whole process of armature detection, including feeding, photographing, preprocessing, discrimination, and sorting, takes less than 3 s. Based on the above reasons, there are various mistakes in manual observation. For example, in the process of workers’ detection, the detection standards are confused, and some minor defects are easy to ignore. As a result, based on the demand of detecting an armature in three seconds, the armatures in the dataset were observed and classified by five workers. After comparing the classification results with the correct classification results of the quality control group, we calculated the manual observation accuracy of each worker and found the average as the manual observation accuracy. In the end, the manual observation accuracy was 91.3%.

### 2.4. Traditional Computer Vision Method

**Figure 5:**
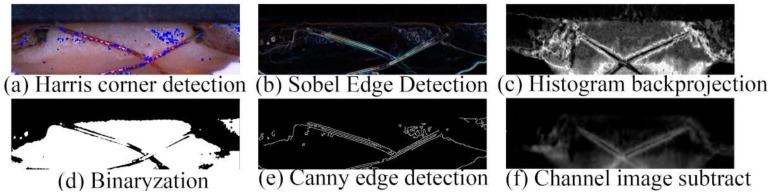
The feature maps extracted by traditional computer vision.

In traditional computer vision, the information such as edges, textures, and colors are extracted and summarized. In our study, we used Sobel edge detection (Figure 5b), binarization (Figure 5d), and Canny edge detection (Figure 5e) to extract edges and textures information. Harris detection was used to detect the corners (Figure 5a). Because the main features of the inspected area were copper wire and tin, we took advantage of histogram backprojection to extract the areas that are similar to the tin in color (Figure 5c). In addition, the copper wire and tin are different in values between the R channel and B channel. Therefore, we subtracted the R and B channels to highlight the position of the copper wire (Figure 5f).

As can be seen from Figure 5, all methods suffer from a degree of information loss due to the effects of areas other than copper wires and tin, so the RGB image keeps the features integrated. We need a more effective feature extraction method for feature extraction of RGB images.

## 3. Methodology

For the armatures in our paper, the defective regions are small, but the features needed are complex and diverse. Furthermore, because of the concentricity error between the armatures and loading platform, the pictures of the armatures have a certain degree of angular difference. Therefore, we need to extract stronger semantic and more abstract features to inspect the surface of the armatures to resist interference. 

### 3.1. ResNet-101 + FPN Network

Thus, we need some way to extract stronger semantic and more abstract image features. A convolutional neural network (CNN) is a kind of backpropagation neural network with deep structure including convolution calculation. It is one of the representative algorithms of deep learning. Generally, CNN has standard structure that the output of the features extractor which consists of stacked convolutional and pooling layers, can be directly input into the classifier. And this typical structure without any additional branch can be called a plain network like Alex, VGG [13,14]. For the plain network, a high-level feature map of each image can be obtained through a deep-network structure. The small features become smaller or even disappear after downsampling. However, the information of high-resolution feature maps without downsampling is not abundant enough. It is not conducive to the extraction and characterization of complex small features, which is required for our dataset. 

**Figure 6:**
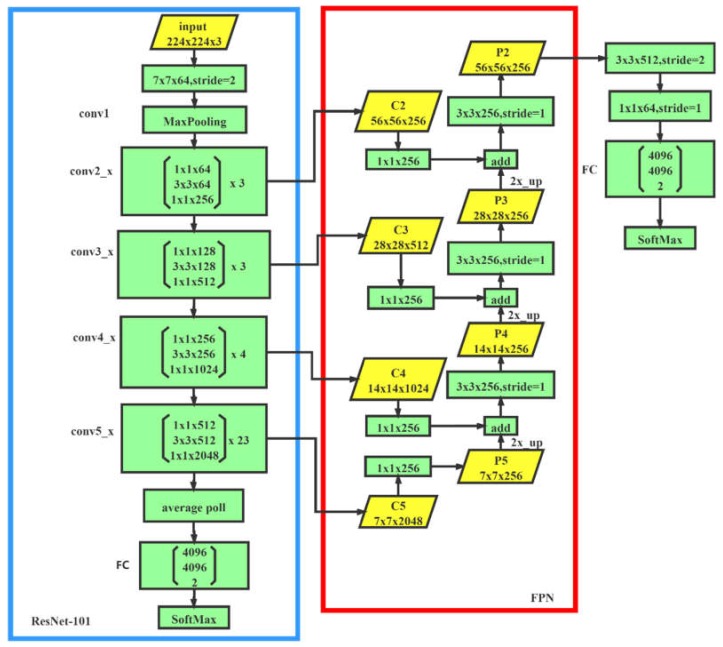
Final network structure of the ResNet101 + feature pyramid network (FPN).

Inspired by the idea of feature pyramids in feature pyramid network (FPN) [22], our novel network as shown in Figure 6 has made some improvements on the basic design of the plain network, which comprises two specific branches: one for extracting feature maps, named the backbone branch, the other used as a network architecture called feature pyramid network (FPN) to fuse feature maps of multiple scales. 

In the process of forward propagation calculation, the backbone branch outputs feature maps of various resolutions. The FPN branch combines low-resolution, semantically strong features with high-resolution, semantically weak features via a top-down pathway, bottom-up pathway, and lateral connections.

Backbone branch: For the classification task, to extract features better, higher layers of representation amplify aspects of the input that are important for discrimination and suppress irrelevant variations [23]. As a result, the network must have enough depth. In order to ensure that the network has enough depth to extract rich semantic information to represent complex features, we used ResNet as the backbone network, since it is connected via a shortcut to learn residual mapping, which is better at transferring gradient information to prevent gradient vanishing and gradient degradation [24,25]. As a result, it can reach thousands of layers compared with a common plain network. In the end, allowing for the time and limited computing resources, we chose ResNet-101, a stack of 101-layer residual units, as our backbone network.

FPN branch: Our goal is to build a feature pyramid with high-level semantics throughout. The construction of our pyramid involves a bottom-up pathway, a top-down pathway, and lateral connections, as introduced in the following. 

The top-down pathway provided the basic feature maps for our FPN. We collected the intermediate feature maps, which is the result of feedforward computation of the backbone network. There are often many layers producing intermediate feature maps of the same resolution, and we say these layers are in the same network stage. In the end, we chose the output of the last layer of each stage as our reference set of feature maps, which we enriched to create our pyramid. This choice is natural, since the deepest layer of each stage should have the strongest features.

For our backbone network, ResNet-101, we used the feature activation output by each stage’s last residual block. We denote the output of these last residual blocks as {C2, C3, C4, C5}, and they have strides of {4, 8, 16, 32} pixels with respect to the input image. We did not include the output of the conv1 in the pyramid due to its large memory footprint. 

The bottom-up pathway outputs higher resolution features by upsampling spatially coarser, but semantically stronger, feature maps from the upper level feature maps collected by the top-down pathway. These features are then enhanced with features from the bottom-up pathway via lateral connections. Each lateral connection merges feature maps of the same spatial size from the bottom-up pathway and the top-down pathway. To start the iteration, we simply attached a 1 × 1 convolutional layer on C5 to produce the coarsest resolution map. Finally, we appended a 3 × 3 convolution to each merged map to generate the final feature map, which was to reduce the aliasing effect of upsampling. This final set of feature maps is called {P2, P3, P4, P5}, corresponding to {C2, C3, C4, C5}, which are respectively of the same spatial sizes.

The result is a feature pyramid that has rich semantics at all levels. The feature pyramid is built quickly from a single input image [22], so P2~P5 all have rich semantics. Additionally, P2 has the highest resolution and fused more features with different scales. As a result, we only took the P2 layer feature map for prediction. We added two convolutional layers after P2 to reduce the size and dimension of the feature map for further information extraction and reduction of computation. After the two convolutional layers, we still added two fully connected layers to summarize the feature information. In the end, we connected SoftMax for binary classification.

### 3.2. Focal Loss

In the case of unbalanced input, the network will converge toward a large quantity of data. It will not care more about the small quantity of data with a large amount of information. For the data in this paper, negative samples have one or more of the above defects. The number of each kind of defective armature is also unbalanced. If the traditional cross-entropy loss function is used, it will lead to poor learning. To solve this problem, the focal loss was proposed by RetinaNet as follows [26]:

(1) $$FL(p_{t})=-\alpha_{t}\left(1-p_{t}{)}^{\gamma }log(p_{t}\right)$$

*α_t_* balances the importance of positive/negative examples. The focusing parameter *γ* smoothly adjusts the rate at which easy examples are down-weighted.

$p\in \;\left[0,1\right]$ is the model’s estimated probability for the class with true label, and *p_t_* is defined as follows:

(2) $$p_{t}=\left\{\begin{array}{c} p,\;\;if\;label\;is\;true\\ 1-p,\;\;otherwise \end{array}\right.$$

We can see from Equation (2) that focal loss adds a modulating factor to the original cross-entropy loss function. When samples are misclassified and *p_t_* is very small, the modulating factor is close to 1, and the loss function has no influence. When *p_t_* approaches 1, the factor goes to 0, and the loss for well-classified examples is down-weighed. Therefore, focal loss can mine unbalanced data and weaken the contribution of the easy-to-classify armatures to loss. In this way, we can better mine the data distribution of negative samples and achieve a better learning effect.

### 3.3. Feature Library Matching

**Figure 7:**
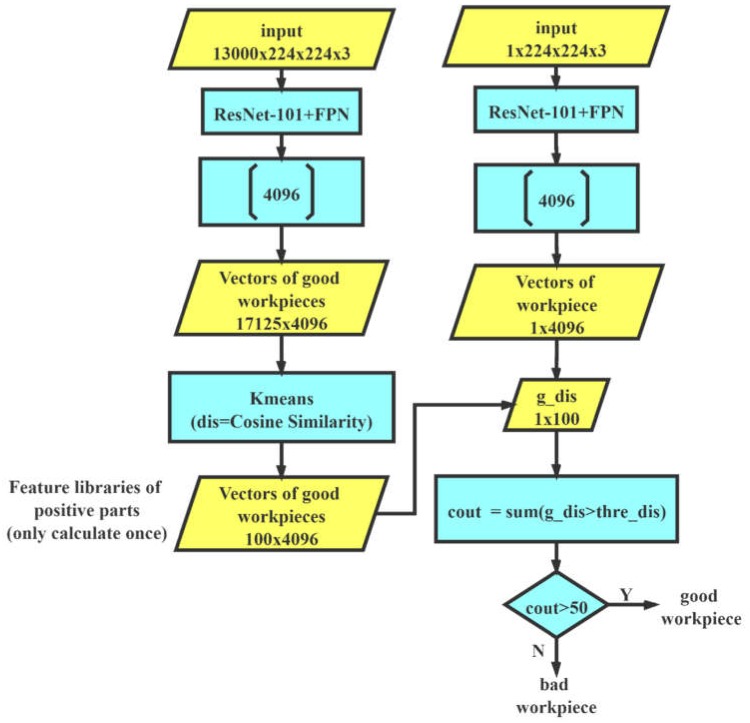
The method of feature library matching.

In the surface detection of the industrial product, the appearance of positive samples is relatively similar, and the appearance of the defective workpieces is relatively different. It is difficult to map all negative samples with different appearances into the same category. Therefore, we took advantage of the previously trained ResNet-101+FPN network, which has a strong representational ability for positive and negative samples, to extract the vector from its end and establish an a priori feature library according to the samples in the training set. We expected to get better results by matching the distance between the sample and a priori feature library to judge the category of the sample. The whole process is shown in Figure 7.

The flat quadrilaterals in the picture represent the data, and the rectangles represent calculations. We used the above-mentioned trained network to infer the training set. Vectors with a size of 4096 extracted from the first fully connected layer were collected and saved. They formed a feature library of the good samples. We inferred the test samples and obtained a feature vector with a size of 4096. We could match the feature vector with the feature library to calculate the similarity between the test samples and the good samples. As a vector similarity measurement, cosine similarity is widely used in text similarity calculation [27] and face recognition [28]. Cosine similarity is defined as follows:

(3) $$similarity=cos(\theta )=\frac{\sum_{i=1}^{n}\left(x_{i}\times y_{i}\right)}{\sqrt{\sum_{i=1}^{n}{\left(x_{i}\right)}^{2}}\times \sqrt{\sum_{i=1}^{n}{\left(y_{i}\right)}^{2}}}$$

*x_i_* and *y_i_* represent the corresponding vectors. The result ranges from −1 to 1. A value of −1 means that the two vectors are pointing in opposite directions, a value of 1 means they are pointing in the same direction, and 0 usually means they are independent. A value in between them indicates similarity or dissimilarity. It can be seen that the range of the values is fixed and will not change like the Euclidean distance, which is helpful for us to find a better threshold value. Equation (3) indicates that it pays more attention to the similarity of the two vectors in the direction, rather than the absolute difference in value. For the comparison of feature vectors, it has a better effect. Therefore, we distinguished the samples by measuring the cosine similarity between feature vectors in the library and feature vectors of the samples.

It would be very time-consuming to compare the cosine similarity between the test workpiece and each vector in the feature library because of the inner product calculation. Therefore, we carried out K-means clustering to cluster the feature libraries of good parts into 100 groups by cosine similarity. The 100 cluster center vectors were selected as a new good feature library, and we only needed to compute the feature library once. We calculated the cosine similarity between the feature vectors of a test armature and each feature vector in the good feature library as *g_dis*, which has 100 dimensions. We set a threshold *thre_dis*. By comparing the size of *g_dis* and *thre_dis*, we can calculate the count, which is the number meeting the threshold condition. If the size of *count* is greater than 50 (the vote was more than half), it will output Y. By the voting mechanism, we could infer whether the sample was good or not.

### 3.4. The Double-Branch Discrimination Mechanism

**Figure 8:**
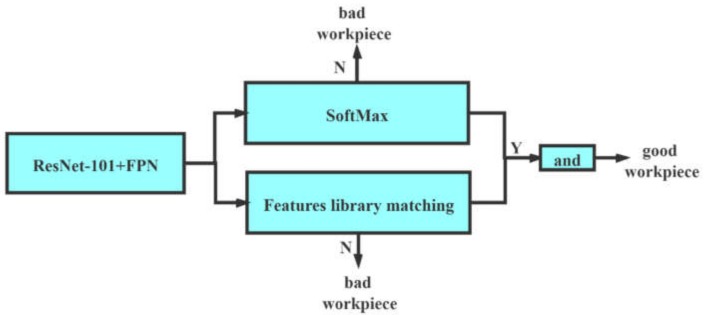
The double-branch discrimination mechanism.

To ensure the quality of the workpiece, it is necessary for mass production to reduce the probability of the defective samples being misjudged as good, so we introduced a double-branch discrimination mechanism to synthesize the results of the two methods. The entire double-branch discrimination mechanism is shown in Figure 8. A branch is determined by the result of the SoftMax layer. The other branch is determined by the method feature library matching. Only when both branches output Y at the same time will the system identify the sample as good. It is equivalent to improving the standard of discrimination. In this way, we expected to achieve better results in defective part discrimination.

## 4. Experiment

### 4.1. Dataset

The image data were captured by the device above. We returned the collected photos to the company’s quality control group. The quality control group divided these photos into two categories according to our classification task. In the rest of this study, we tested and validated our approach based on this dataset and its classification. Because there are many types of defective armatures and the distribution is uneven, we tried and trained on different data volumes. We found that the larger the quantity of data, the more robust the final model is. To ensure a good learning effect for the data distribution of the defective samples and improve the robustness of the model, we studied a large quantity of data. Finally, the training set contained 26,000, and the test set contained 11,106. The ratio of the number between the training set and the test set is approximately 7:3, which followed the common ratio of 7:3 in machine learning. 

### 4.2. Implementation Details

Data Augmentation: To fully connect the layers and enhance robustness, we resized the images to 240 × 240 × 3 pixels and randomly cropped them to 224 × 224 × 3 pixels as the input in each batch. Moreover, in the training process, we randomly flipped and rotated the loaded data and adjusted the brightness. To prevent the small changes in image quality making the judgment of the network change, we finally normalized the input data [29].

Experiment Environment: We built the network environment through TensorFlow, a deep learning framework compatible with Windows. We created a graph model and trained on a 1080 Ti graphics card with 12 GB video memory.

Training Strategy: Because the shortcut connection in the residual network can help the information to propagate easily [25,30], when the structure of FPN was added, the network became more complex, and information was harder to backpropagate. Thus, the method of training a backbone network, a side-branch structure, and an up-sampling structure together cannot ensure that the backbone network contains good semantic information for FPN to extract. To obtain a better backbone network and train our network better, we used a step-by-step method of training. 

### 4.3. Training and Results

**Figure 9:**
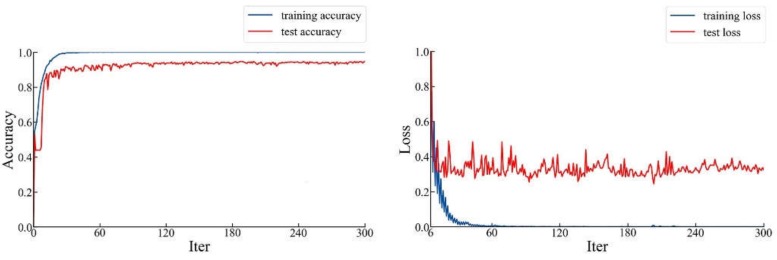
The accuracy and loss curve of the ResNet-101+FPN transfer learning from the ImageNet classification model.

Firstly, we trained our network directly. The initial learning rate was set to 0.001 and was attenuated 10 times after the 80th epoch. In order to prevent the results from falling into a local optimal solution and gradient oscillation [31], we used the momentum optimizer to optimize loss and update the weight. We set the momentum to 0.01. After 300 iterations, we obtained the curve of loss and accuracy as the number of iterations increased, as shown in Figure 9.

As can be seen in Figure 9, the network tends to converge, and the highest accuracy in the whole process was 96.5%, while the loss was 0.317. Compared with ResNet-101, the accuracy was significantly improved.

The model with the highest accuracy on the test set was saved. Then, we tested it on test set and obtained the confusion matrix as shown in Table 2.

**Figure 10:**
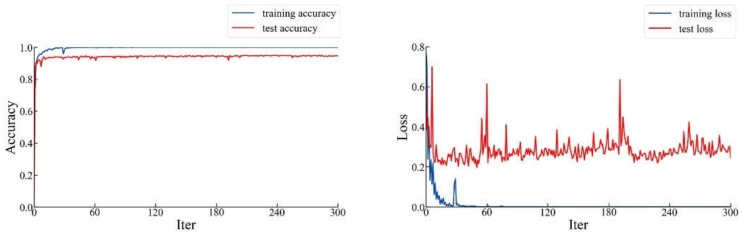
The accuracy and loss curve of the ResNet-101 initialized by trained the model of ResNet-101+FPN.

Secondly, we extracted the partial parameters of ResNet-101 from the above trained ResNet101+FPN. We used the parameters to initialize and trained the ResNet-101 to obtain a more expressive backbone network. After 300 iterations, we obtained the curve of loss and accuracy as the number of iterations increased, as shown in Figure 10. 

As can be seen from Figure 10, the highest prediction accuracy in the whole process was 96.8%, while the loss was 0.213. Compared with ResNet-101 transfer learning from the ImageNet classification model, our initialization method significantly improved the performance of ResNet-101. We realized the idea of training a good backbone network. We saved the model that performed best in accuracy to use in the follow-up work.

**Figure 11:**
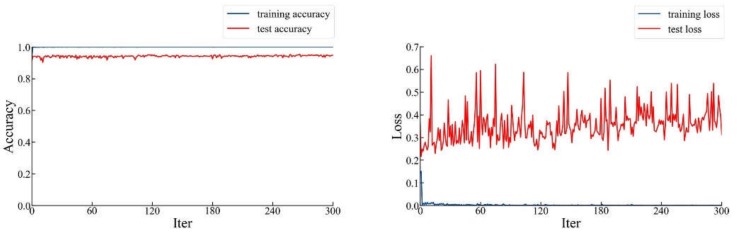
The accuracy and loss curve of the ResNet-101+FPN in the first step.

In the end, we used the trained backbone network to train ResNet101-FPN in two steps. In the first step, we used the more expressive ResNet-101 model above as the model of the backbone network. We adjusted the learning rate of ResNet-101 layers to 0 to fix the weight parameters of each layer. In this way, we only trained related layers of FPN. After 300 iterations, we obtained the curve of loss and accuracy as the number of iterations increased, as shown in Figure 11.

As can be seen in Figure 11, the highest prediction accuracy in the whole process is 97.2 %, while the loss was 0.293.

**Figure 12:**
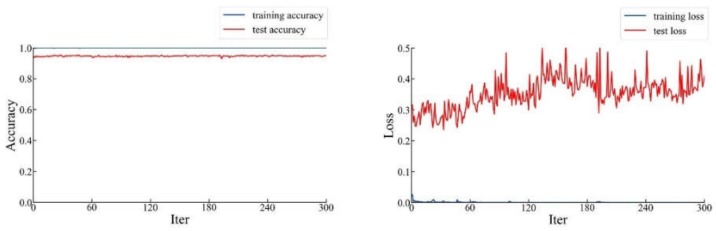
The accuracy and loss curve of the ResNet-101+FPN in the second step.

In the second step, we loaded the parameters obtained in the previous step into our network. We trained the entire network including ResNet-101 and FPN. After 300 iterations, we obtained the curve of loss and accuracy as the number of iterations increased, as shown in Figure 12. 

As can be seen from Figure 12, the highest prediction accuracy in the whole process was 97.1%, while the loss was 0.192. In the first step of training, the accuracy increased by 0.1% compared with the result of training our network directly. Although the accuracy did not increase again in the second step, the loss decreased significantly, indicating that the overall effect of the network was improved to some extent. Therefore, compared with directly training ResNet-101+FPN, our distributed training method achieved a significant improvement in terms of accuracy and loss.

The model with the highest accuracy on the test set was saved. Then, we tested to get the confusion matrix shown in Table 3.

Through the above steps, we obtained a backbone network with a strong capability for feature extraction and a multi-scale fusion network. This was also better than directly training our network.

### 4.4. Establishing the Double-Branch Discrimination System and Results

Above all, we obtained a good network model. Since the range of cosine similarity is between -1 and 1, we searched the *thre_dis* with the step size of 0.05 and verified the branch of feature library matching on the test set to find the best threshold. Finally, when the *thre_dis* is 0.35, this branch has the highest accuracy in the test. The accuracy is 97.3%. After testing on the test set, we obtained the confusion matrix shown in Table 4. 

It can be seen that the method of creating a priori feature library can improve the accuracy.

To further reduce the number of the defective samples being misjudged as good, we combined the two branches in the way described before. Then, we tested the test set to get the confusion matrix shown in Table 5.

The accuracy of the test set dropped from 97.2% to 97.1%. As can be seen from Table 3, 4074 armatures were rejected by the first branch. Among them, 3956 were rejected correctly, and 118 were rejected incorrectly. As can be seen from Table 4, 4075 armatures were rejected by the second branch. Among them, 3969 were rejected correctly, and 106 were rejected incorrectly. As can be seen from Table 5, after combining the above two branches, 4147 were rejected. Among them, 4003 were rejected correctly, and 144 were rejected incorrectly. Through comparison, we find that more negative simples were correctly identified after combining the above two branches. Since both branches are required to be Y at the same time, the identification standards for good parts become stricter. This can guarantee better quality of the armatures.

### 4.5. Comparison and Discussions

The evaluation indexes used in this paper are accuracy, precision, recall, and f1-score, commonly used for classification problems. Our paper mainly considered the comparison of accuracy and precision. The accuracy can reflect the overall performance of the model. The accuracy rate can reflect the proportion of negative samples that are misjudged as positive samples, which is very important to ensure the quality of industrial products.

We trained and tested the same dataset on the Support Vector Machine (SVM), a machine-learning model, and the deep-learning model mentioned in our paper. Table 6 provides a comprehensive comparison of the index.

In summary, from Table 6, the armatures detection method we propose achieved good results. Compared with other structures, the structure of ResNet-101+FPN we designed is improved in all aspects. We also propose a training method to make our network performance better. 

We can see that the proposed feature library matching method improved in all respects based on the parameters of our classification network. Although the accuracy and recall rate were reduced by 0.1%, the precision was improved by 0.5% after merging the two branches. It is a significant improvement in precision for the mass production under the condition that the accuracy is almost unchanged. With the precision improved, the probability of the defective samples being misjudged as good reduced, which ensured the quality of the workpiece and was in line with the requirements proposed by the enterprise. From the perspective of the time, there is little difference between the time of feature library matching and the time of the double-branch discrimination mechanism. Therefore, we chose double-branch discrimination mechanism to be applied to the production line.

### 4.6. Validation in Actual Production

In order to validate the reliability of the discrimination system in actual production, we inspected the armatures produced by the company in one day. The total number of the armatures inspected was about 360,000. Among them, the proportion of defective parts was about 2%. Due to the imbalance of positive and negative workpieces and most of the armatures with relatively good surfaces on the production line, the accuracy reached 98.9% in the actual production line. The result was also calculated by comparing the classification result of the discrimination system with the classification result of the quality control group. The whole process of the detection of the armatures, including feeding, photographing, preprocessing, discrimination, and sorting, is automated and takes less than 3 s, which is the enterprise’s required time to identify an armature. Therefore, the armature inspection system proposed in this paper can meet the requirements of enterprise, both in terms of detection accuracy and detection time. The discrimination system can be stably used in the armature production line.

## 5. Conclusions

In this paper, a novel and complete system based on computer vision and deep learning is proposed for surface inspection of armatures in vibration motors with miniature volume. Concerning the characteristics of the data samples, we designed the structure of ResNet-101+FPN, which uses ResNet-101 as the backbone network and integrates the idea of FPN structure to utilize its representation capacity more effectively for small features. The network achieved satisfying performance on our dataset by accurately predicting the categories of armatures. In addition, we proposed a training method to make the network designed by us perform better. To guarantee the quality of the motor, we had to reduce the proportion of the defective samples being identified as good. Therefore, based on the trained network, we proposed a feature library matching method. After combining the classification network and the feature library matching method, the miscalculation of defective parts was significantly decreased. When testing on the production line, the discrimination could also ensure robustness and universality. The whole process of the detection of the armature takes less than 3 s, which is the enterprise’s required time to identify an armature. Therefore, the system proposed in this paper can meet the requirements of enterprise, both in terms of effect and detection time. The discrimination system can be stably used in the armature production line.

## Figures and Tables

**Table 1:** Types of surface defects.

Terminal exposed (Figure 4a)	The terminal connecting the copper wire and the resistor is not covered with tin. It is not strong enough to ensure the service life.
Rheostat bonded tin (Figure 4b)	A long piece of tin is attached to the rheostat, which will affect the use of the motor.
Too thin cropper wire (Figure 4c)	Because of the high temperature, the copper wire connecting the terminals is thinned, which will cause the copper wire to break easily.
Copper wire stuck, long tin (Figure 4d)	The attached tin on the copper wire is too long. The tin is not as hard as the copper wire, which will also cause the copper wire to break easily.
Foreign matter (Figure 4e)	There are foreign bodies sticking in this area, which will cause noise in the process of rotation.
Tin bead (Figure 4f)	In the process of rotation, the tin beads easily fall off, which will have a serious impact on the service life.

**Table 2:** The confusion matrix of the ResNet-101+FPN model (one stage).

		Predicted Value		Total
		Positive	Negative	
**Observed Value**	Positive	6788	153	6941
	Negative	221	3944	4165

**Table 3:** The confusion matrix of the ResNet-101+FPN model (two stage).

		Predicted Value		Total
		Positive	Negative	
**Observed Value**	Positive	6823	118	6941
	Negative	209	3956	4165

**Table 4:** The confusion matrix of the branch implemented by feature library matching.

		Predicted Value		Total
		Positive	Negative	
**Observed Value**	Positive	6835	106	6941
	Negative	196	3969	4165

**Table 5:** The confusion matrix of the double-branch discrimination mechanism.

		Predicted Value		Total
		Positive	Negative	
**Observed Value**	Positive	6797	144	6941
	Negative	162	4003	4165

**Table 6:** A comprehensive comparison of the index.

	Accuracy	Recall	Precision	F1-Score	Time Per Image/s
SVM	83.2%	85.3%	87.5%	86.4%	0.574
VGG-16	92.4%	93.3%	94.6%	93.9%	0.035
ResNet-101	93.6%	95.1%	95.0%	95.0%	0.084
ResNet-101+FPN (trained directly)	96.6%	97.8%	96.8%	97.3%	0.095
ResNet-101+FPN (two stage trained)	97.1%	98.3%	97.0%	97.6%	0.095
Feature library matching	97.3%	98.5%	97.2%	97.8%	0.186
Double-branch discrimination mechanism	97.2%	97.9%	97.7%	97.7%	0.205
